# The extent of public awareness and use of the Global Solar UV Index as a worldwide health promotion instrument to improve sun protection: A systematic review and meta‐analysis

**DOI:** 10.1111/php.14028

**Published:** 2024-10-14

**Authors:** Isabelle Kaiser, Annette B. Pfahlberg, Maria Lehmann, Esther Buchta, Wolfgang Uter, Olaf Gefeller

**Affiliations:** ^1^ Institute of Medical Informatics, Biometry, and Epidemiology Friedrich‐Alexander University Erlangen‐Nürnberg Erlangen Germany

**Keywords:** awareness, meta‐analysis, sun protection, systematic review, use, UV Index

## Abstract

Thirty years ago, the Global Solar UV Index (UVI) has been introduced as a health promotion instrument to improve sun protection. We assessed systematically global levels of awareness and use of the UVI as a prerequisite for the preventive effectiveness of this public health tool. We conducted a comprehensive literature search across 10 databases, including PubMed, Scopus and Web of Science Core Collection, as well as clinical trial registries and gray literature databases. The risk of bias of studies was evaluated using the Joanna Briggs Institute checklist for prevalence studies. In addition to narrative and descriptive analysis, we performed meta‐analyses with geographical subgroup analyses to statistically summarize the results. In total, we identified 40 publications from 39 different studies across multiple global regions. However, the number of studies in the analyses varies depending on the outcome. The results, especially the awareness of the UVI, were largely dependent on the specific geographical location of the studies. While the prevalence of awareness of the UVI is high among Australian populations, there is considerable variability in levels of awareness across other global regions. At the same time, the use of the UVI is at a low level across all regions, demonstrating the need for enhanced dissemination of knowledge about the perils associated with ultraviolet radiation and the advantages of using the UVI.

## Graphical abstract

The Global Solar Ultraviolet Index (UVI) was introduced by the WHO and partner organizations in 1995 as a simple measure of the intensity of solar UV radiation, providing guidance for the population to use appropriate sun protective measures. This systematic review assessed global levels of awareness and use of the UVI. While there is considerable variability in levels of awareness across global regions, the use of the UVI is at a low level across all regions, demonstrating the need for enhanced dissemination of knowledge about the perils associated with ultraviolet radiation and the advantages of using the UVI.

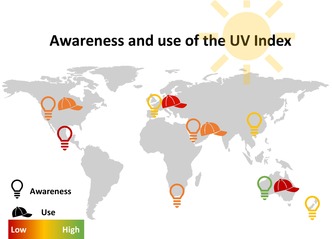

**Abbreviations**
ICNIRP: International Commission on Non‐Ionizing Radiation Protection
JBI: Joanna Briggs Institute
ROB: risk of bias
UNEP: United Nations Environment Programme
UV: ultraviolet
UVI: ultraviolet index
WHO: World Health Organization
WMO: World Meteorological Organization

## INTRODUCTION

Solar ultraviolet (UV) radiation is classified into three subtypes depending on the wavelength of this electromagnetic radiation: UV‐A (315–400 nm), UV‐B (280–315 nm), and UV‐C (100–280 nm).^1^ Due to absorption and scattering processes in the atmosphere, only UV‐A and roughly 10% of UV‐B reach the earth's surface and thus exhibit biological effects on humans.^2^ While exposure to UV radiation has positive health effects, most importantly vitamin D production in humans, the negative health consequences of overexposure overshadow the risk‐benefit assessment.^3^ The International Agency for Research on Cancer has classified UV radiation as group I human carcinogen, that is, based on strong evidence from numerous experimental and epidemiologic studies regarding several different types of skin cancer.^4^ UV radiation has also been epidemiologically linked to cataract development, while animal and in vitro studies have proven a causal relationship.^5^ Both adverse health effects, skin cancer and cataract, have a large public health impact worldwide, thus demanding for effective sun protection measures.^6^, ^7^

Humans lack a sensory organ for UV radiation. The correlation between temperature and intensity of UV radiation arriving at the earth's surface is too weak to use temperature as a reasonable proxy. To find the appropriate level of sun protection intuitively is therefore challenging. Terrestrial UV radiation is measured by spectroradiometers operated at various stations being part of country‐specific networks,^8^ and information about actual and forecasted UV levels needs to be visualized and disseminated to the public.^9^ Thirty years ago, the Global Solar UV Index (UVI) has been developed as a worldwide health promotion instrument to improve sun protection.^10^ The UVI constitutes a measure of the daily maximum intensity of erythemally weighted solar UV irradiance or, in other words, the potential of the prevailing UV radiation to induce sunburn.^11^ In 1992, a precursor of the current UVI was developed in Canada. Three years later, the World Health Organization (WHO), together with the World Meteorological Organization (WMO), the International Commission on Non‐Ionizing Radiation Protection (ICNIRP), and the United Nations Environment Programme (UNEP), adopted a slightly modified version of the Canadian original and promoted it by publishing a practical guide for the public.^12^ The practical guide proposed a harmonized UVI reporting scheme and attached preventive recommendations to the different UVI categories. At a “low” UVI level (UVI values, always reported as integer values, <3), no protection is required. For “moderate”^3^, ^4^, ^5^ and “high”^6^, ^7^ UVI values, application of all sun protection measures including shade (during midday hours), clothing, sunscreen, sunglasses, and a hat is recommended. At “very high”^8^, ^9^, ^10^ or “extreme” (11+) UVI levels, people should use all of the aforementioned sun protection measures, seek shade all day, and, in addition, avoid being outdoors during midday hours.

Numerous epidemiologic surveys over the last 30 years have addressed awareness and use of the UVI in different populations worldwide. Two previous systematic reviews have provided an overview of the literature until 2010^13^ and 2017,^14^ respectively. The objective of our investigation is to update these reviews providing an in‐depth comparison of study findings for all studies on the topic that have been published over the last decades and, as a supplement facilitating the communication about UVI dissemination on the population level, to summarize their findings in different regions of the world in a quantitative meta‐analysis, to the extent the heterogeneity of assessing UVI‐related information in the individual studies allows such a quantitative synthesis.

## MATERIALS AND METHODS

We developed a study protocol that was published^15^ and registered with PROSPERO (registration number: CRD42018093693). Also part of the study protocol was the topic of how the public understands the UVI, which was intentionally omitted from this report. The systematic review is reported according to the PRISMA guideline.^16^

### Eligibility criteria

The eligibility criteria were defined following the PICOS scheme,^17^ see Table 1. Only studies that used the definition of the UVI as defined by the WHO and its partner organizations in 1995 were included. Regarding the study design, we considered only quantitative studies, including both experimental and observational studies. In case of intervention studies (randomized or non‐randomized), only data at baseline were extracted, as this represents the state of knowledge of the participants before the intervention and reflects the “status quo ante” of the study population. There was no restriction on the language, but the studies had to have at least an English or German abstract. For the full‐text screening, potentially important studies in other languages than English or German were translated using Google Translator.

**TABLE 1:** Eligibility criteria according to the PICOS scheme.

Criteria	Inclusion	Exclusion
Participants	No restraints regarding the type of study population	None
Intervention	The Global Solar UVI as introduced in 1995 by the WHO and partner organizations^18^	Other versions of UV indices
Comparator	Not applicable	Not applicable
Outcome	Quantitative estimates of awareness and use of the UVI	Other outcomes
Study design	Only quantitative studies including experimental studies (randomized controlled trials) and observational studies (cross‐sectional studies, case‐control studies, before‐and‐after studies with and without controls, prospective and retrospective cohort studies)	Qualitative studies

### Search process

For the systematic search, we first conducted an electronic literature search across 10 different databases, including PubMed/Medline, Scopus/Embase, Web of Science Core Collection, ScienceDirect, The Cochrane library (CDSR, CENTRAL, CMR, DARE, HTA, EED), Applied Sociological Sciences Index and Abstracts (ASSIA), EPPI‐Centre database of health promotion research (DoPHER, Bibliomap, TRoPHI), Educational Resource Information Centre database (ERIC), Sociological Abstracts, and PsycINFO. We also searched the two clinical trials registries ClinicalTrials.gov and the International Clinical Trials Registry Platform (ICTRP) for relevant studies. Furthermore, we explored the gray literature through the OpenGrey and Bielefeld Academic Search Engine (BASE) databases to identify relevant studies that were not published in peer‐reviewed journals. In addition, we employed the forward snowballing technique on six studies relevant to this topic. These include five previous epidemiological studies^19^, ^20^, ^21^, ^22^, ^23^ on different aspects of the UVI published between 1997 and 2004, and the systematic review on the effectiveness of the UVI as a health promotion instrument conducted by Italia and Rehfuess published in 2012.^13^ Forward snowballing is an efficient search approach that investigates citations to specific reference papers and thus looks forward in time when performing a search among citations.^24^ We used the Scopus database for this purpose. Additionally, the references of the studies identified through the electronic searches that met our inclusion criteria were manually searched for relevant studies. Additionally, we assessed all studies included in two previous systematic reviews^13^, ^14^ on the same topic for eligibility.

The search terms for the electronic database search consist of variations of the term “UV index” and a diverse range of terms associated with the outcomes awareness, knowledge, and use of the UVI for sun protection. The search string for searching the Scopus database can be found in Table S1. For the remaining electronic databases, the search string was adapted to the requirements of the respective database. We conducted the search in August 2022 and updated it in June 2023. The search results were limited to publications that were published after 1995 since the concept of the UVI was first introduced in this year.

### Study selection

The screening and selection process implemented to include all eligible studies consisted of two phases. After importing the search results into Endnote X9 and removing duplicates, the titles and abstracts of all identified publications were evaluated for eligibility. This was done independently by two reviewers. All studies deemed potentially eligible by at least one reviewer was included in the full‐text screening. Thus, only studies that were judged as not relevant by both reviewers were excluded, which ensures high sensitivity of the search procedure. In the second phase, two reviewers independently assessed the eligibility of the full‐text publications. Discrepancies in decisions were resolved by discussion and, in case of sustained disagreement, by involving an independent third reviewer.

### Data extraction

The data extraction was performed independently by two reviewers, and discrepancies were resolved by discussion involving an independent third reviewer. The following information was extracted for each study: period of data collection, study region, study setting and method of participant selection, study design, primary focus, sample size, data collection instrument, and details of outcome assessment. The primary focus of the studies was assigned to three categories. The category “UVI” comprises studies in which the investigation of awareness and/or use of the UVI was the main topic. The category “Sun protection incl. UVI” includes studies in which the focus was on measures of sun protection, with the UVI being an integral component of these measures. The category “Other focus” comprises studies with a different focus, in which the survey of awareness or use of the UVI was only marginally. We defined subcategories for the outcomes “awareness of the UVI” and “use of the UVI” to reduce structural heterogeneity within studies of the same outcome category, as both terms are highly generic and cover different ways of assessing the corresponding outcome information, see Table 2. For the outcome “awareness,” we distinguished between three subcategories. The first subcategory concerns the general awareness of the existence of the concept of the UVI, that is, whether respondents have ever heard of the term UVI. The second subcategory pertains to awareness of the existence of UV forecasts, for example, in the newspaper or TV, irrespective of whether respondents actually consult them. The third subcategory, “Awareness of the UVI on a specific day,” comprises questions on whether respondents have heard or seen the UVI forecast on a specific day. Regarding the outcome “use,” we defined two subcategories: The first subcategory comprises the active use of the UVI for the purpose of personal sun protection. The second subcategory encompasses actively seeking information about the UVI value or checking the UVI. Studies contributing data on the latter outcome subcategory did not contain a clear indication that the UVI information is used for sun protection purposes. The outcomes extracted from the studies were assigned to these subcategories. Furthermore, frequency measures (mostly percentages) describing the degree of awareness and use of the UVI were extracted.

**TABLE 2:** Subcategories of the outcomes “awareness of the UVI” and “use of the UVI.”

Awareness of the UVI	Use of the UVI
Awareness of existence of the UVI Awareness of existence of the UV forecast Awareness of the UVI on a specific day	Active use of the UVI for sun protection Seeking information about UVI (behavioral impact unclear)

### Risk of bias assessment

The risk of bias (ROB) of all studies selected for inclusion in the systematic review was assessed independently by two reviewers using the Joanna Briggs Institute (JBI) Checklist for Prevalence Studies.^25^ The JBI checklist was developed by a methodological JBI working group in Australia for the ROB assessment of studies reporting prevalence and cumulative incidence information. It includes nine items relating to participant selection, assessment of information, study conduct, and analysis methods that are either answered with yes or no, depending on whether the item was met or not. If the information reported in the study was too sparse, the item was rated unclear. The tool also provides the option “not applicable” if an item could not be applied to a study. Based on the ratings of the nine items, the reviewers assigned an overall ROB rating to each study. The JBI tool does not provide an algorithm for determining the study's overall ROB rating based on a scoring system or similar. The overall ROB rating is based on a joint appraisal of all relevant aspects of the study, with low methodological study quality in key aspects not being compensated for by other components of the study in which a high methodological quality was present. The evaluation process is inevitably prone to include subjective elements. To establish a consistent rating standard, the reviewers thoroughly reviewed and discussed each item of the JBI tool beforehand while establishing specific decision rules for determining the overall ROB. The overall ROB was either rated low, high, or unclear.

In case of discrepant ROB ratings, consensus meetings consisting of three reviewers were held to discuss the disagreements and derive at a consensus decision.

### Statistical analysis

The main characteristics of the included studies, their findings, and their ROB ratings are presented in summary tables. Forest plots, created for each subcategory of the outcome, show the reported proportion and its corresponding confidence interval (CI) for each study. If the study did not report the confidence interval, we calculated it using the Wilson method, provided that we could extract the sample size to which the reported proportion refers. Studies reporting no quantitative results or providing vague quantifications (like, e.g., “less than 20%”) were excluded from this analysis. If a study comprised multiple independent cross‐sectional surveys, their results were considered separately in the analysis.

Statistical pooling of results through meta‐analysis was performed for outcome subcategories with reasonably homogeneous definitions of outcomes. To further reduce structural heterogeneity, we only considered population‐based studies for meta‐analysis and performed subgroup analyses by study region. Pooled prevalence estimates and corresponding 95% CIs were calculated using a generalized linear mixed model (GLMM) with random effects. The between‐study variance *τ* ^2^ was estimated using the maximum‐likelihood estimator. Heterogeneity between studies was assessed by calculating the *I* ^2^‐statistic, which ranges from 0% to 100% with *I* ^2^ > =75% indicating considerable heterogeneity.^26^ Furthermore, sensitivity analyses excluding studies with high or unclear ROB were performed when possible. Analyses were conducted in R version 4.2.2 and R packages “meta” and “metafor.”

## RESULTS

### Search results

**FIGURE 1:**
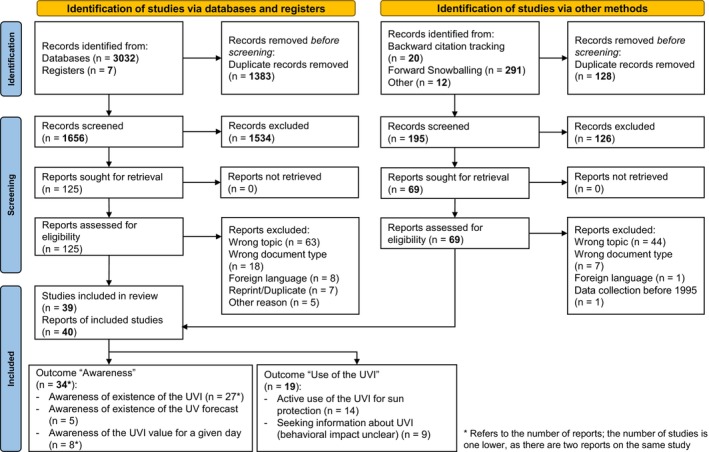
PRISMA flowchart of the systematic literature review process.

As depicted in the PRISMA flow chart (Figure 1), the literature search in the electronic databases and registers yielded a total of 3039 references. After elimination of duplicates, 1656 publications remained for the title and abstract screening. Of 125 publications that were included in the full‐text screening, 101 were excluded as they did not fulfill our eligibility criteria. Thus, 24 publications were eligible for inclusion. Through backward citation tracking, forward snowballing, review of the studies included in Ref.^13^, ^14^, and expert consultation, an additional 323 references were identified. Removal of duplicates resulted in 195 references that were screened for eligibility. After the full‐text screening, 16 studies remained. The main reasons for exclusion in the full‐text screenings were wrong outcomes and wrong document types. Nine studies were published in foreign languages that could not be translated using Google Translator with sufficient quality to enable an assessment of the studies eligibility. Altogether, 39 studies published in 40 reports met the inclusion criteria and were included in our review. The number of studies is one less than the number of reports, as two publications^22^, ^27^ refer to the same study from New Zealand. However, Reeder et al.^27^ only analyzed a subsample of the Bulliard et al.^22^ study population, so we included both publications.

### Study characteristics

Thirty‐four studies examined the awareness of the UVI. Of these, 26 studies in 27 publications explored the general awareness of the UVI, while five studies focused on awareness of the UV forecast, and eight studies investigated the awareness of the UVI on a specific day. The use of the UVI was investigated by 19 of the 39 studies. Among them, 14 studies assessed the active use of the UVI for sun protection, and nine studies investigated the active seeking of UVI information without inquiring about any consequences on the subjects' personal sun protection behavior. Study characteristics of the included studies are shown in Table 3.

**TABLE 3:** Study characteristics of all included studies (*N* = 39) and publications (*N* = 40).

Author (publication year)	Study region	Period of data collection	Study setting/study design	Primary focus	Sample size	Outcomes assessed				
						Awareness			Use	
						Awareness of existence of UVI	Aware‐ness of existence of UV‐Forecast	Aware‐ness of UVI on specific day	Active use for sun protection	Seeking information about UVI
*Australia*										
Kricker et al. (1997)^28^	New South Wales, Australia	1997	General population/Cross‐sectional	UVI	*N* = 504		x		x	
White et al. (1997)^29^	Melbourne, Victoria, Australia	January 1997	General population/Cross‐sectional	UVI	*N* = 486		x	x		
Alberink et al. (2000)^19^	Queensland, Australia	April–October 1997	General population/Cross‐sectional	UVI	*N* = 977		x			
Blunden et al. (2004)^20^	Perth, Western Australia, Australia	December 1999	General population/Cross‐sectional	UVI	*N* = 501	x		x		
Makin et al. (2007)^30^	Melbourne, Victoria, Australia	March 2002	General population/Cross‐sectional	UVI	*N* = 419		x	x		
Harrison et al. (2007)^31^	Queensland, Australia	June 2002	Directors of day care centre/Cross‐sectional	Sun protection incl. UVI	*N* = 1383	x				
Carter et al. (2007)^32^	Perth, Western Australia, Australia	January 2005	General population/Cross‐sectional	UVI	*N* = 404			x		
Mair et al. (2012)^33^	Queensland, Australia	December 2009–January 2010	General population/Cross‐sectional	Sun protection incl. UVI	*N* = 141	x				
Thomas et al. (2017)^34^	Sydney, New South Wales, Australia	2015	Healthcare Facilities/Cross‐sectional	Sun protection incl. UVI	*N* = 661	x			x	
Scott et al. (2021)^35^	Australia	March 2019	University students/Before‐and‐after intervention study without controls	Sun protection incl. UVI	*N* = 161				x	x
Scott et al. (2021)^36^	Western Australia, Australia	March 2019	University students/Cross‐sectional	Sun protection incl. UVI	*N* = 275				x	x
*New Zealand*										
Bulliard et al. (2001)^22^	New Zealand	January 1999	General population/Cross‐sectional	UVI	*N* = 396	x				
Reeder et al. (2001)^27^	New Zealand	January 1999	General population restricted to Maori ethnicity/Cross‐sectional	Sun protection incl. UVI	*N* = 57^a^					
McGee et al. (2002)^37^	Dunedin & Hawkes Bay, New Zealand	December 1998–February 2000	General population/Repeated cross‐sectional	Sun protection incl. UVI	1998: *N* = 116 1999: *N* = 115 2000: *N* = 141			x		
*North America*										
Geller et al. (1997)^23^	USA	September 1995	General population/Cross‐sectional	UVI	*N* = 700	x		x		
Purdue et al. (2001)^38^	Canada	September 1996	General population/Cross‐sectional	Other focus	*N* = 4023	x				
McCarthy et al. (1999)^39^	Galveston Island, USA	July 1997	General population/Cross‐sectional	Sun protection incl. UVI	*N* = 55	x				
Patlola et al. (2023)^40^	USA	January 2023	General population/Cross‐sectional	Other focus	*N* = 200					x
*Europe*										
Bais et al. (1997)^41^	Greece	Spring 1997	General population/Cross‐sectional	Other focus	*N* = 572					x
Wester et al. (2000)^42^	Sweden	September 1997	General population/Cross‐sectional	UVI	*N* = 1094	x				
Government Statistical Service (2000)^43^	UK	September 1999	General population/Cross‐sectional	UVI	*N* = 1869	x				
Sécurité Solaire (2000)^44^	France	January 2000	General population/Cross‐sectional	UVI	*N* = 1004	x				
Bränström et al. (2003)^21^	Sweden (Stockholm County)	2001	General population/Randomized controlled trial	Sun protection incl. UVI	Unclear				x	
Morris et al. (2011)^45^	Devon and Cornwall, UK	August – September 2002	General population/Cross‐sectional	UVI	*N* = 466	x		x		
Unverricht et al. (2007)^46^	Dresden, Saxony, Germany	2007	Outdoor workers/Cross‐sectional	Sun protection incl. UVI	*N* = 128		x			
Börner et al. (2010)^47^	Germany	May–June 2007	General population/Cross‐sectional	UVI	*N* = 1501	x			x	
Diffey et al. (2009)^48^	UK	May–September	2007	General population/Cross‐sectional	Sun protection incl. UVI	*N* = 2061			x	
Krebs et al. (2008)^49^	Suisse	September 2002–2008 (annual survey waves)	General population/Repeated cross‐sectional	UVI	2002: *N* = 822 2003: *N* = 920 2005: *N* = 917 2006: *N* = 905 2008: *N* = 1007	x				x
Klostermann et al. (2014)^50^	Bavaria, Germany	2010–2011	Day care centre (school entry examination)/Cross‐sectional	Sun protection incl. UVI	*N* = 4579	x			x	
Hault et al. (2016)^51^	Dresden, Saxony, Germany	June 2011	Outdoor workers/Cross‐sectional	Sun protection incl. UVI	*N* = 40	x				
Sin et al. (2013)^52^	Paris, France	December 2011	Healthcare Facilities (dermatologists)/Cross‐sectional	UVI	*N* = 165	x			x	x
Capellaro et al. (2015)^53^	Germany	August 2013	General population/Cross‐sectional	Other focus	*N* = 4000	x				
Gefeller et al. (2022)^54^	Germany	April – September 2016	Directors of day care centre/Cross‐sectional	UVI	*N* = 436	x			x	
Busuttil et al. (2019)^55^	Malta	NA	General population/Cross‐sectional	UVI	*N* = 400				x	x
*Saudi Arabia*										
Sultana et al. (2020)^56^	Saudi Arabia and Bahrain	January 2018 – May 2019	General population/Cross‐sectional	Sun protection incl. UVI	*N* = 830	x			x	
Addas et al. (2021)^57^	Saudi Arabia	October 2004 – December 2020	University staff and students/Cross‐sectional	Other focus	*N* = 542	x				x
*Others*										
Wright et al. (2014)^58^	South Africa	August – October 2012	Day care centre/Cross‐sectional	Sun protection incl. UVI	*N* = 707	x				
Gao et al. (2014)^59^	Shenyang, China	October – Fall 2013	Physicians and medical students/Cross‐sectional	Sun protection incl. UVI	*N* = 385	x				x
Aryal et al. (2018)^60^	Nepal	March–October 2016	Police facilities/Cross‐sectional	Sun protection incl. UVI	*N* = 265	x				
Morales‐Sánchez et al. (2021)^61^	Mexico	2020	Students (16–18 years old), general population/Cross‐sectional	Other focus	*N* = 1368 (Students: *N* = 748, Adults: *N* = 620)	x				x

*Note*: Studies are grouped by study region and, within each region, sorted according to the period of data collection.

Abbreviation: UVI, Ultraviolet Index.

^a^ Subgroup of the study sample already described in Bulliard et al.^22^

Eleven studies were located in Australia and two in New Zealand. Four studies were conducted in North America, three in the United States, and one in Canada. Additionally, we found 16 European studies from seven different countries, including six studies from Germany and three from the United Kingdom. Furthermore, two studies were from Saudi Arabia and one study from each of the following countries: South Africa, China, Nepal, and Mexico. The data collection periods specified by the studies cover almost the entire period since the introduction of the UVI concept in 1995. The first study to examine the awareness of the UVI of the population was that of Geller et al. who conducted surveys in 58 cities in 50 states of the United States in September 1995, immediately after the UVI had been introduced.

The majority of studies (*n* = 26) addressed the general population, while the remaining studies addressed certain subgroups of the population like students, directors of child care centers, or certain occupational groups. The investigation of the awareness and/or use of UVI was the main objective of 17 publications. The same number of publications focussed primarily on sun protection measures, with the UVI being described as an integral component of these measures. The remaining six publications had a different focus and only marginally addressed awareness or use of the UVI. Details on study aims for all studies can be found in Table S2. The majority of studies were one‐time cross‐sectional surveys (*n* = 35). Two studies were interventions studies, of which we only used baseline values for analysis. Furthermore, two studies^37^, ^49^ consisted of several independent cross‐sectional surveys at different points in time. McGee et al.^37^ interviewed parents about sun protection at selected beaches in New Zealand in the summers of 1998, 1999, and 2000. As part of a telephone survey on sun exposure and sun protection behavior of the Swiss population, the study by Krebs et al.^49^ also surveyed the awareness and use of the UVI every year from 2002 to 2008 (except 2004 and 2007).

Temporal analyses within regions for the various outcomes were not feasible, as either the number of studies conducted over the period under consideration was insufficient or the heterogeneity between studies was too substantial to allow comparison of results. For example, the three North American studies investigating awareness of the existence of the UVI were conducted over a period of just 2 years between September 1995 and July 1997. Consequently, no conclusions could be drawn about a possible temporal trend. In other regions, such as Europe, where the number of existing studies was higher and the data collection periods were distributed over a much longer time span (from 1997 to 2019), the heterogeneity of the studies, for example, regarding the target population, was too large to enable comparison of results. Figure S1 illustrates the temporal distribution of the studies, stratified by outcome and region.

### Awareness of the UVI

*Awareness of existence of the UVI*: Details on the assessment of the awareness of existence of the UVI, as well as reported results of the 26 studies, are presented in Table 4. The majority of studies assessed “awareness” as ever having heard of the UVI. In three studies, the exact wording of the question was unclear. The most common question types were closed questions with dichotomous (yes/no) response options and those with multiple answer options. Regarding the ROB assessment using the JBI tool, 10 studies were assessed as having a low ROB, 11 as having a high ROB, and five as having an unclear ROB.

**TABLE 4:** Details on data collection instrument and outcome assessment, as well as reported results for the outcome “Awareness of existence of the UVI.”

Author (publication year)	Data collection and type of question	Question	Results	ROB
*Australia*				
Blunden et al. (2004)^20^	Telephone survey; Closed question	“Have you heard of the term UV index?”	90% (*n* = 452) affirmed the question	Low
Harrison et al. (2007)^31^	Postal questionnaire; Closed question	“Heard of the UV index”	92.8% of the participants (*N* = 1383) affirmed the question	Low
Mair et al. (2012)^33^	Online questionnaire; Closed question	“Ever heard of the UV index”	92% of the participants affirmed the question	High
Thomas et al. (2017)^34^	Unclear; Closed question	“Do you know what the UV index is?”	93% (*n* = 548) affirmed the question	High
*New Zealand*				
Bulliard et al. (2001)^22^	Telephone survey; Closed question	“Aware of the UVI”	43% of the participants affirmed the question	Low
Reeder et al. (2001)^27^	Telephone survey; Closed question	“Have you seen or heard anything about the Ultra Violet Index?”	≈33% (*n* = 19) affirmed the question	Low
*North America*				
Geller et al. (1997)^23^	Telephone survey; Closed question	“Heard of the UVI”/ “aware of UVI”	63.6% (*n* = 445) affirmed the question	Low
Purdue et al. (2001)^38^	Telephone survey; Question with multiple answer options	“Awareness of UV Index”	53.1% (*n* = 2116) answered “always/often”	Low
McCarthy et al. (1999)^39^	Personal interview; Unclear type of question	“Aware of UV radiation indices or warnings”	5.5% (*n* = 3) affirmed the question	High
*Europe*				
Wester et al. (2000)^42^	Telephone survey; Closed question	“Have you seen or heard of the UV index?”	27% of the participants affirmed the question	Unclear
Government Statistical Service (2000)^43^	Personal interview; Closed question	“Have you ever read or heard of [the Solar UV Index]”	64% of the participants affirmed the question	Low
Sécurité Solaire (2000)^44^	Telephone survey; Closed question	“Have you ever read, seen or heard the term UVI or solar index last summer or in the last few years?” (original question: “Avez‐vous déjà lu, vu ou entendu au cours de l'été dernier ou des précédentes années le terme Index UV, Indice UV, ou Indice Solaire?”)	74% (*n* = 738) affirmed the question	Unclear
Morris et al. (2011)^45^	Personal interview; Question with multiple answer options	“Heard of the UVI”	67% (*n* = 314) affirmed the question	High
Börner et al. (2010)^47^	Telephone survey; Closed question	“Do you know the UV‐index?”	27% (*n* = 405) affirmed the question	Low
Krebs et al. (2008)^49^	Telephone survey; Closed question	“Do you know what the UV index is?” (original question: “Wissen Sie, was der UV‐Index bedeutet?”, related to awareness)	2002: 37% (*n* = 304) 2003: 34% (*n* = 314) 2005: 43% (*n* = 391) 2006: 38% (*n* = 349) 2008: 38% (*n* = 385)	Low
Klostermann et al. (2014)^50^	Unclear	“UV index is not known”	26.2% (*n* = 1157) did not know the UVI ➔ 73.8% know the UVI	Unclear
Hault et al. (2016)^51^	Unclear	“Heard of the ultraviolet index (UVI)”	30% of the participants affirmed the question	High
Sin et al. (2013)^52^	Postal questionnaire; Closed question	“Have you ever heard of the UV index of Météo‐France?” (original question: “Avez‐vous entendu parler de l'indice UV diffusé par Météo‐France?”)	80% (*n* = 132) affirmed the question	High
Capellaro et al. (2015)^53^	Telephone survey; Question with multiple answer options	“Have you ever heard of or read about an UV‐Index in any form?”	29.5% of the participants affirmed the question	Low
Gefeller et al. (2022)^54^	Personal interview; Closed question	“Have you ever heard of the UV index?”	47.7% (*n* = 208), affirmed the question	High
Busuttil et al. (2019)^55^	Telephone survey; Unclear type of question	Unclear	96% of the participants are aware of the UVI	Unclear
Saudi Arabia				
Sultana et al. (2020)^56^	Online questionnaire; Closed question	“Aware about UV Index”	30.6% of the participants are aware of the UVI	High
Addas et al. (2021)^57^	Postal questionnaire; Unclear type of question	Unclear	29% of the participants were already aware of the UVI	High
*Others*				
Wright et al. (2014)^58^	Postal questionnaire; Closed question	“Seen or heard about the UVI”	28.8% (*n* = 203) affirmed the question	Low
Gao et al. (2014)^59^	Unclear; Question with multiple answer options	Response categories “I have heard of it, but don't know the exact meaning” and “Yes” of the question “Do you know the meaning of UVI?”	53.2% (*n* = 205^a^) affirmed the question	High
Aryal et al. (2018)^60^	Postal questionnaire; Closed question	“Knowledge of UVR and UVI”	24.9% affirmed the question	Unclear
Morales‐Sánchez et al. (2021)^61^	Postal questionnaire; Unclear type of question	Unclear	3.4% of the adults know the UVI	High

*Note*: Studies are grouped by study region and, within each region, sorted according to the period of data collection, following the same order as in Table 3.

Abbreviation: UVI, Ultraviolet Index.

^a^ The proportion of participants who know the UVI was calculated by combining the two response categories “I have heard of it, but don't know the exact meaning” (*N* = 186) and “Yes” (*N* = 19) to the question “Do you know the meaning of UVI?”.

The reported proportions of awareness among the study participants were very heterogenous. The four Australian studies indicated a high awareness of UVI (90%–93%), whereas in the two publications of the study originating from New Zealand, the reported proportion were much lower (33% and 43%, respectively). The reported proportions in the studies conducted in North America ranged from 5.5% to 63.6% and in the 12 European studies from 27% to 96%. The highest proportion of study participants who are aware of the UVI was reported by a Maltesian study (96%, 95% CI 93.6–97.5). However, the methodological quality of the study is unclear, as the associated gray publication only consisted of a poster with limited information. The lowest proportion (3.4%, 95% CI 2.2–5.1) was reported in a study by Morales Sánchez et al.^61^ in which adults were interviewed at a dermatological center in Mexico. A forest plot showing the reported proportions of all 27 publications can be found in Figure S2.

**FIGURE 2:**
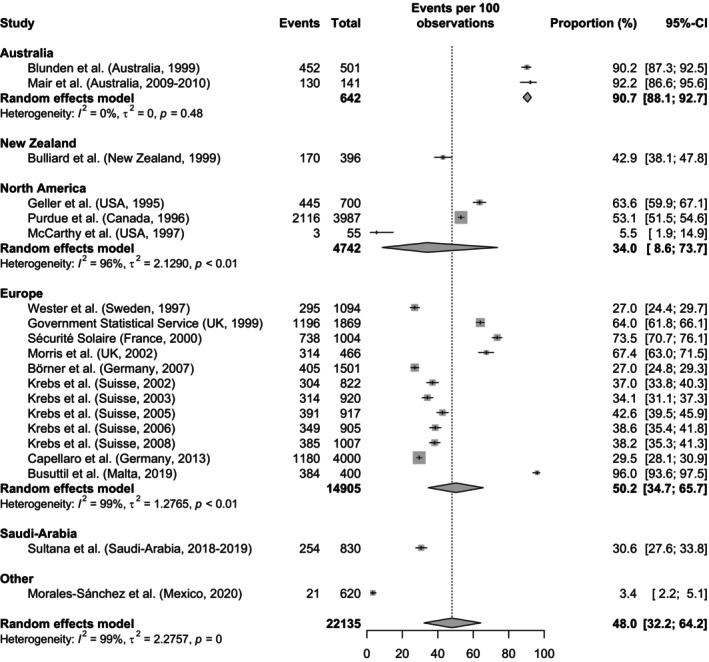
Forest plot with meta‐analysis showing the proportions of the general awareness of the UVI grouped by study region. Within each region, the studies are sorted according to the period of data collection following the same order as in Table 3. The size of the box is proportional to the sample size.

The results of the meta‐analysis restricted to population‐based studies are shown in Figure 2. Ten of the 26 studies were excluded, as they were conducted in special subpopulations which cannot be assumed to be representative for the general population. The study of Reeder et al.^27^ was also excluded from meta‐analysis as the study sample is a subsample of Bulliard et al.^22^ The overall pooled proportion from the random effects model for all regions is 48.0% (95% CI 32.2–64.2), with a significant evidence of between‐study heterogeneity (*I* ^2^ = 99%, *τ* ^2^ = 2.28, *p* < 0.001). When assessed by region, Australian studies had the highest estimate of UV awareness (90.7%, 95% CI 88.1–92.7) with low heterogeneity between studies (*I* ^2^ = 0%, *τ* ^2^ = 0, *p* = 0.48). In contrast, in studies conducted in North America and Europe, the pooled proportions are 34.0% (95% CI 8.6–73.7, *I* ^2^ = 96%, *τ* ^2^ = 2.13, *p* < 0.01) and 50.2% (95% CI 34.7–65.7, *I* ^2^ = 99%, *τ* ^2^ = 1.28, *p* < 0.01), respectively. The sensitivity analysis, which excluded studies with a high or unclear ROB, yielded a similar value for the overall pooled proportion (47.6%, 95% CI 36.1–59.3, *I* ^2^ = 99%, *τ* ^2^ = 0.6938, p < 0.01). However, notable differences emerged for the pooled estimates for the two regions, North America (58.1%, 95% CI 50.7–65.2) and Europe (38.6%, 95% CI 31.5–46.2). Results of the sensitivity analysis are shown in Figure S3.

**FIGURE 3:**
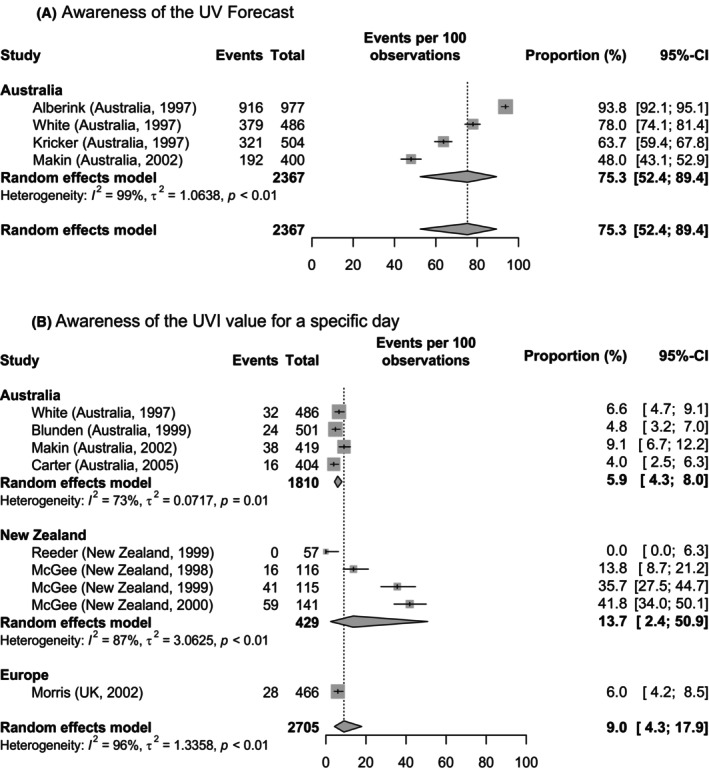
Forest plots with meta‐analysis showing the proportions of (A) the awareness of the UV forecast and (B) the awareness of the UVI value for a specific day grouped by study region. Within each region, the studies are sorted according to the period of data collection following the same order as in Table 3. The size of the box is proportional to the sample size.

*Awareness of existence of the UV forecast*: Five studies assessed the awareness of existence of the UV forecast, see Subtable 1 of Table 5. Four out of the five studies were located in Australia, while only one study was conducted in a European country. Most of the studies assessed the awareness of the UV forecast by asking if the UV forecast/UV alert had been seen or heard. The study by Unverricht et al.^46^ indirectly assessed UV forecast awareness by asking “Do you know the indication ‘UVI’ from the weather report?”. None of the studies was rated with a low ROB in the ROB assessment, as three studies received a high ROB and two studies received an unclear ROB rating. The reported proportion of study participants who are aware of the UV forecast ranges from less than 20%^46^ to 93.8%.^19^ The study by Unverricht et al.^46^ was excluded from the meta‐analysis due to the imprecise proportion reported in its results (“<20%”). Consequently, only the four Australian studies were taken into account for the meta‐analysis, see Figure 3A. The pooled proportion of awareness of the UV forecast from meta‐analysis yielded an overall 74.9% (95%CI: 51.3–89.4) with high level of between‐study heterogeneity (*I* ^2^ = 99%, *τ* ^2^ = 1.12, *p* < 0.01). A sensitivity analysis was not performed for this outcome due to the lack of studies assessed as having a low ROB.

**TABLE 5:** Details on data collection instrument and outcome assessment, as well as reported results for the sub‐outcomes “Awareness of existence of the UV forecast” and “Awareness of the UVI on a specific day.”

Author (publication year)	Data collection and type of question	Question	Results	ROB
Subtable 1: Awareness of the UV forecast				
*Australia*				
Kricker et al. (1997)^28^	Unclear	“UV forecast or measurement had been seen or heard”	64% (*n* = 321) affirmed the question	Unclear
White et al. (1997)^29^	Telephone survey; Closed question	“Had heard or seen reports or forecasts about UVR in the weather reports”	78% of the participants affirmed the question	Unclear
Alberink et al. (2000)^19^	Postal questionnaire; Closed question	“Reported seeing or hearing the UV forecast”	93.8% (*n* = 916) have seen or heard of the UVI over the year Summer: 89% (*n* = 867) have seen or heard of the UVI Winter: 86% (*n* = 837) have seen or heard of the UVI	High
Makin et al. (2007)^30^	Personal interview; Question with multiple answer options;	“Recalling having ever seen the UV Alert”	48% of the 400 participants have ever seen the UV Alert	High
*Europe*				
Unverricht et al. (2007)^46^	Unclear Closed question	“Do you know the indication ‘UV index’ from the weather report” (original question: “Kennen Sie die Angabe “UV‐Index” aus dem Wetterbericht”)	<20% of participants affirmed the questionn	High
Subtable 2: Awareness of the UVI on a specific day				
*Australia*				
White et al. (1997)^29^	Telephone survey; Closed question	“Had seen the UVR forecast for the Saturday of the previous week‐end” “Had seen the UVR forecast on Sunday of the previous week‐end”	6.6% (*n* = 32) of all participants had seen the UVR forecast for Saturday. 4.9% (*n* = 24) of all participants had seen the UVR forecast for Sunday.	Unclear
Blunden et al. (2004)^20^	Telephone survey Closed question Open question	“Did you notice today's UV level?” “What was the UV index level for today?”	5% (*n* = 24) noticed today's UV level 3.3% (*n* = 16) knew the correct UV index level for today. This corresponds to 80% of the 24 people who had noticed the UVI prediction	Low
Makin et al. (2007)^30^	Personal interview; Question with multiple answer options, open question	“Do you know the forecast UV levels for today?” “Seeing the [UV] Alert on the day of the survey”	9% of 419 participants knew the correct answer. 6% of 400 participants have seen the UV Alert on the day of the survey	High
Carter et al. (2007)^32^	Personal interview; Unclear question type, open questions	“Aware of the UVI forecast for the day of the interview” “What was the forecast today for the UVI” “What is the average UVI in Perth for winter/summer?”	4% (*n* = 16) were aware of the UVI forecast on that day. 0.5% (*n* = 2), knew the correct UVI forecast for today. 5.2% (*n* = 21), knew the average UVI in Perth for winter. 8.2% (*n* = 33), knew the average UVI in Perth for summer	High
*New Zealand*				
Bulliard et al. (2001)^22^	Telephone survey Unclear question type	“Did you see or hear the UVI for your area on Sunday?”	No quantitative results	Low
Reeder et al. (2001)^27^	Telephone survey; Unclear question type;	“Did you see or hear the UV Index for your area on Sunday?”	“None could recall seeing or hearing the UVI for their area on the most recent Sunday” (=0%)	
McGee et al. (2002)^37^	Personal interview; Closed question	“Knowledge of burn‐time and/or the UV Index (UVI) for the day”	1998: 13.9% of 116 participants 1999: 35.7% of 115 participants 2000: 41.8% of 141 participants	High
*Europe*				
Morris et al. (2011)^45^	Personal interview; Unclear question type	“Knew the UVI value on the day of the survey”	6% (=28 of 466) knew the UVI value on the day of the survey	High

*Note*: Studies are grouped by study region and, within each region, sorted according to the period of data collection, following the same order as in Table 3.

Abbreviations: UVI, Ultraviolet Index; UVR, ultraviolet radiation.

*Awareness of the UVI on a specific day*: The awareness of the UVI value for a specific day was assessed by seven studies and reported in eight publications, as Bulliard et al.^22^ and Reeder^27^ refer to the same study. Details on the outcome assessment and reported results are shown in Subtable 2 of Table 5. Four studies were conducted in Australia, two in New Zealand, and one in the United Kingdom. Some studies asked the study participants if they had seen the UV forecast or noticed the UV level on the day of the survey,^20^, ^30^, ^32^, ^37^, ^45^ while others asked for the awareness of the UV forecast on the previous week‐end.^22^, ^27^, ^29^ With the exception of McGee et al.,^37^ all reported proportions are below 10%. In the three survey waves of McGee et al.,^37^ the percentage of those who knew the UVI for the day of the survey increased from 13.8% in 1998 to 41.8% in 2000. However, the study assessed knowledge of the UVI together with knowledge of burn‐time, so the results may be biased. The ROB was rated as low in only two of the seven studies.^20^, ^22^ Four studies were rated as having a high ROB^30^, ^32^, ^37^, ^45^ and one study as having an unclear ROB.^29^ Some studies^20^, ^30^, ^32^ additionally asked for the actual or forecasted UVI value for the day of the survey in open questions, but a maximum of 9% of participants answered correctly.

Results from meta‐analysis are shown in Figure 3B. The study of Bulliard et al.^22^ was excluded as they did not report any quantitative results. Pooled proportion of the Australian studies was 5.9% (95% CI: 4.3–8.0) with low heterogeneity between studies (*I* ^2^ = 12%, *τ* ^2^ = 0, *p* = 0.33). The studies from New Zealand yielded a pooled proportion of 13.7% (95% CI: 2.4–50.9; *I* ^2^ = 87%, *τ* ^2^ = 3.06, *p* < 0.01). The overall pooled proportion from random effects model was 9.0% (95% CI: 4.3–17.9, *I* ^2^ = 96%, *τ* ^2^ = 1.34, *p* < 0.01). A sensitivity analysis was not performed for this outcome due to the limited number of studies assessed as having a low ROB.

### Use of the UVI

Due to the heterogeneous and often unclear nature of the questions asked for the outcome “use of the UVI,” we limited our analysis to narrative and descriptive analyses, without conducting any meta‐analysis.

**FIGURE 4:**
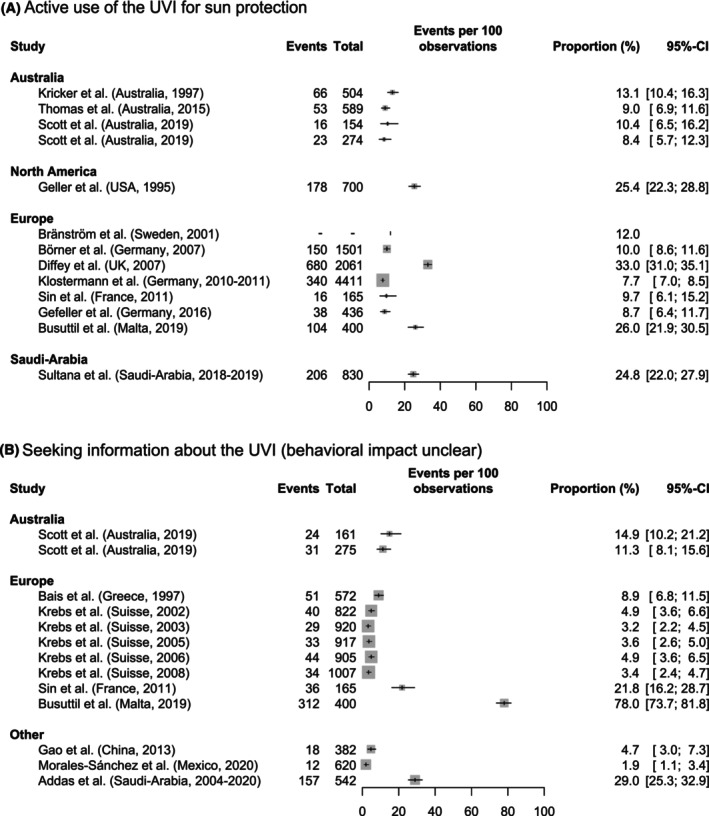
Forest plots showing the proportions of (A) the outcome “Active use of the UVI for sun protection” and (B) the outcome “Seeking information about the UVI (behavioral impact unclear).” Studies are grouped by study region and, within each region, sorted according to the period of data collection, following the same order as in Table 3. The size of the box is proportional to the sample size.

*Active use of the UVI for sun protection*: The active use of the UVI with impact on the sun protection behavior was examined in 14 studies, see Table 6. Four studies have been conducted in Australia, two in North America, seven in Europe, and one in Saudi Arabia. The studies were conducted between 1995^23^ and 2019.^35^, ^36^, ^55^, ^56^ In the ROB assessment, only two studies received a low ROB,^23^, ^47^ four were rated with an unclear ROB^21^, ^28^, ^50^, ^55^ and eight were rated with a high ROB.^34^, ^35^, ^36^, ^40^, ^48^, ^52^, ^54^, ^56^ Figure 4A shows the reported proportions from all studies except for Patlola et al.^40^ who provided no quantitative results. Comparable proportions, ranging from 8% to 13%, were consistently found in the four Australian studies. The European studies reported more heterogenous results ranging from 8% to 33%. The lowest proportion (7.7%, 95% CI 7.0–8.5) of individuals who actively use the UVI for adjusting their sun protection behavior accordingly was reported by a German study^50^ in which parents of children aged 5–6 years were surveyed. The highest proportion (33.0%, 95% CI 31.0–35.1) was reported by Diffey et al.^48^ However, in contrast to the other studies they did not assess a regular use. Instead, they asked for “using the UV index at least once or twice.”

**TABLE 6:** Details on data collection instrument and outcome assessment, as well as reported results for the outcome “Active use of the UVI for sun protection.”

Author (Publication year)	Data collection and type of question	Question	Results	ROB
*Australia*				
Kricker et al. (1997)^28^	Unclear	“Used the UV information […] last summer to decide about sun protection”	13% of the population reported a “regular use” 75% of the population sample reported that they rarely used the UV information on half or more of the weekends last summer to decide about sun protection.	Unclear
Thomas et al. (2017)^34^	Unclear; Closed question	Answer “Yes, and I use it regularly” of the question “Do you know what the UV index is?”	9% (*n* = 53) use the UVI regularly.	High
Scott et al. (2021)^35^	Online questionnaire; 5‐Point Likert Scale	“I use the UVI as a tool to protect me from the sun”	Never: *n* = 50 (32.5%) Rarely: *n* = 47 (30.5%) Sometimes: *n* = 41 (26.6%) Often: *n* = 13 (8.4%) Everyday: *n* = 3 (1.9%)	High
Scott et al. (2021)^36^	Online questionnaire; 5‐Point Likert Scale	“Use of the UV index as a sun protection tool”	Never: *n* = 97 (35.3%) Rarely: *n* = 90 (32.7%) Sometimes: *n* = 64 (23.3%) Often: *n* = 19 (6.9%) Everyday: *n* = 4 (1.5%)	High
*North America*				
Geller et al. (1997)^23^	Telephone survey; Closed question	“Used it to determine the best time to tan”	25.4% of the 700 participants confirmed this statement.	Low
Patlola et al. (2023)^40^	Online questionnaire; Unclear type of question	Unclear	No quantitative information, but “the use of UV index numbers to make an informed decision (*p* = 0.0284) were significantly associated with more frequent sunscreen usage”	High
*Europe*				
Bränström et al. (2003)^21^	Postal questionnaire; Unclear type of question	“Use of the UV index as presented in the news media”	12% of the participants affirmed the question	Unclear
Börner et al. (2010)^47^	Telephone survey; Question with multiple answer options	“Do you consider the UV‐index for personal sun exposure and sun protection behavior?”	17% (*n* = 255) of all participants state that they consider the UV index information for their sun exposure and protection behavior; 10% (*n* = 150) of all participants consider UV‐index information more frequently and alter [their] personal sun exposure and sun protection behavior accordingly	Low
Diffey et al. (2009)^48^	Online questionnaire; Question with multiple answer options	“Using the UV index at least once or twice to plan their sun exposure”	33% of respondents reported using the UVI at least once or twice to plan their sun exposure	High
Klostermann et al. (2014)^50^	Unclear; Question with multiple answer options	“Use of the UV index”	7.7% (*n* = 340) affirmed the question	Unclear
Sin et al. (2013)^52^	Postal questionnaire; Closed question	“In your sun protection advice, do you rely on the UV index?” (original question: “Dans vos conseils de prevention solaire, vous appuyez‐vous sur l'indice UV?”)	9.7% of total participants gave the answers “often” or “always”	High
Gefeller et al. (2022)^54^	Personal Interview; Closed question	“Does the actual UVI‐value influence sun protection at your kindergarten?”	8.7% (*n* = 38) of the participants confirmed that the actual UVI influences sun protective measures at their kindergartens	High
Busuttil et al. (2019)^55^	Unclear	“Do you consider the UV index for personal sun exposure and sun protection behavior?”	26% claimed that UVI forecasts impacted on their work/ leisure activities	Unclear
*Other*				
Sultana et al. (2020)^56^	Online questionnaire; Closed question	“Consider UV index for personal sun exposure sun protection behavior”	24.8% (*n* = 206) consider the UVI for their personal sun exposure and sun protection	High

*Note:* Studies are grouped by study region and, within each region, sorted according to the period of data collection, following the same order as in Table 3 Abbreviation: UVI, Ultraviolet Index.

*Seeking information about the UVI (behavioral impact unclear)*: Nine studies assessed the active seeking information about the UVI irrespective of its impact on sun protective behavior. Study characteristics are shown in Table 7. The studies primarily originate from Australia (*n* = 2) and Europe (*n* = 4), with additional contributions from China (*n* = 1), Mexico (*n* = 1), and Saudi Arabia (*n* = 1). “Seeking information about the UVI” in this context is mostly assessed as “checking the UVI” or “attending the UVI forecasts.” The forest plot in Figure 4B displays the reported proportion for all studies. The proportion of individuals who actively look for UVI information ranges from 2% (95% CI: 0.01–0.03) to 78% (95% CI: 0.74–0.82), with most studies at the lower end of the range. Krebs et al.^49^ conducted annual surveys between 2002 and 2008 with constant low proportions between 3% and 4%. According to the study of Busuttil et al.,^55^ 78% of the Maltesian population follow the UVI forecast in summer, which significantly exceeds the proportions reported in the other studies. However, the study was rated with an unclear ROB due to limited information, as was the study by Bais et al.^41^ Only the study of Krebs et al.^49^ was rated with a low ROB, and the remaining six studies^35^, ^36^, ^52^, ^57^, ^59^, ^61^ were rated as having a high ROB.

**TABLE 7:** Details on data collection instrument and outcome assessment, as well as reported results for the outcome “Seeking information about the UVI (behavioral impact unclear).”

Author (publication year)	Data collection and type of question	Question	Results	ROB
*Australia*				
Scott et al. (2021)^35^	Online questionnaire; 5‐Point Likert Scale	“I check the UVI”	Checking the UVI: Never: *n* = 44 (28.6%) Rarely: *n* = 43 (27.9%) Sometimes: *n* = 43 (27.9%) Often: *n* = 21; (13.6%) Everyday: *n* = 3 (1.9%)	High
Scott et al. (2021)^36^	Online questionnaire; 5‐Point Likert Scale	“I check the UV index”	Checking the UVI: Never: *n* = 83 (30.2%) Rarely: *n* = 91 (33.1%) Sometimes: *n* = 69 (25.1%) Often: *n* = 26 (9.5%) Everyday: *n* = 5 (1.8%)	High
*Europe*				
Bais et al. (1997)^41^	Unclear type of question	“Attend […] the UV forecasts”	9% of the participants answered “regularly” 60% of the participants answered “occasionally”	Unclear
Krebs et al. (2008)^49^	Telephone survey; Closed question	“Did you pay special attention to the UVI or consciously inform yourself about it” (original question: “Haben Sie den UV‐Index speziell beachtet oder sich bewusst danach informiert”)	2002: 4.9% of all participants 2003: 3.2% of all participants 2005: 3.6% of all participants 2006: 4.9% of all participants 2008: 3.4% of all participants	Low
Sin et al. (2013)^52^	Postal questionnaire; Closed question	“In the past year, did you voluntarily inform yourself at least once about the UVI?” (original question: “Au cours de l'année précédente, vous êtes‐vous volontairement informés au moins une fois sur l'IUV?”)	21.8% (*n* = 36) affirmed the question.	High
Busuttil et al. (2019)^55^	Telephone survey; Unclear type of question	Unclear	78% follow UVI forecasts in summer	Unclear
*Other*				
Gao et al. (2014)^59^	Unclear; Question with multiple answer options	“Paid attention to UVI when reading the weather forecast”	Always: *n* = 4 (1.0%) Often: *n* = 14 (3.7%) Sometimes: *n* = 81 (21.2%) Seldom: *n* = 195 (51.0%) Never: *n* = 88 (2.1%)	High
Morales‐Sánchez et al. (2021)^61^	Postal questionnaire; Unclear type of question	Unclear	1.9% of adults consult the UVI daily	High
Addas et al. (2021)^57^	Postal questionnaire; Unclear type of question	Unclear	29% of participants were aware of the index and followed it	High

*Note*: Studies are grouped by study region and, within each region, sorted according to the period of data collection, following the same order as in Table 3.

Abbreviation: UVI, Ultraviolet Index.

## DISCUSSION

Although the Global Solar UVI was introduced almost 30 years ago in 1995 by the WHO and partner organizations as a public health tool to inform the public about health risks of UV radiation and to change peoples' sun protection behavior, our systematic review revealed a mixed picture regarding awareness and a very limited use of the UVI. We identified 40 publications from 39 different studies in most parts of the world reporting very heterogeneous results depending primarily on the study region.

Our results show that there exist clear differences between the countries in terms of awareness of the UVI. The highest level of awareness can be found in Australia, where over 90% of Australian study participants were aware of the UVI. This is not unexpected, given the long history of skin cancer prevention campaigns in Australia. Due to its geographical location and predominantly fair‐skinned population, Australia has the highest incidence of melanoma worldwide. To counteract the increasing trend of new melanoma cases, the country introduced the SunSmart program in 1988.^62^ Community programs and mass media campaigns were designed to influence attitude, knowledge, and behavior related to sun protection among the Australian population. Even earlier, in 1980, the Cancer Council Victoria began to raise awareness of the dangers of UV radiation. The program also focused on raising awareness among children and implementing sun protection measures in schools and early childhood services. Now, 97% of early childhood services and 90% of primary schools in the Australian state of Victoria have implemented a written sun protection policy.^63^ Although New Zealand has also high UV radiation levels and skin cancer rates, there is a notable discrepancy to Australia in the general awareness of the UVI. Only 43% of those surveyed (and only one third in the Maori subgroup^27^) were aware of the UVI.^22^

In the rest of the world, population awareness of the UVI is low to moderate with pooled proportions ranging from 34% in North America (95% CI 8.6–73.7) to 50.2% in Europe (95% CI 34.7–65.7). However, when individual studies are considered, the results are highly heterogeneous. On the one hand, there are studies that report percentages similar to those in Australia, such as the Maltesian study by Busuttil et al.^55^ (96%). On the other hand, there are also studies in which the proportion of study participants who are aware about the UVI is very low (North America: 5.5%, Mexico: 3.4%).

Different target populations limit the comparability of individual study results. For example, the study by Sin et al.^52^ surveyed dermatologists in health care facilities who, by virtue of their profession, would be expected to have a higher prevalence of UVI knowledge than the general population. In addition, the methodological quality of the studies is often insufficient, which limits the reliability of study results. Only 10 of the 26 studies that investigated the general awareness of the existence of the UVI and only two of the seven studies that investigated the awareness of the UVI on a specific day were rated with a low ROB. All other studies received a high or unclear ROB rating due to methodological flaws or missing information. This is especially true for studies in the lower and upper ranges of the reported proportions. Our sensitivity analysis showed that studies with a high or unclear ROB strongly bias the results of our meta‐analysis at the regional level. However, the direction of bias was not uniform. The pooled proportion for North America showed a downward bias due to studies with high or unclear ROB. Conversely, for Europe, the pooled proportion exhibited an upward bias. In other regions, the number of studies with low ROB is insufficient to permit any conclusion regarding the potential for and direction of bias.

In contrast to awareness of the UVI, active use of the UVI is at a low level in all study regions. This applies to both subcategories of this outcome, active use for sun protection and active seeking of UVI information (which may not translate into adequate UV‐related behavior). It is alarming that although more than 90% of the Australian population are aware of the UVI, less than 10% use it for adapting their sun protective behavior. In the past, UVI values were disseminated primarily through the newspaper, which had limited accessibility and may explain the observed low use in earlier studies. Nowadays, however, the UVI is integrated in many weather apps and weather forecasts, which considerably facilitates accessibility. Yet, current studies also show that use of the UVI remains at a low level. Possible reasons are the perception of a lacking personal relevance and a contradictory motivation aimed at increasing attractiveness through tanning. More efforts are needed to communicate not only the UVI values themselves, but also the recommendations regarding sun protection behavior that are associated with the different UVI values.^20^ The intervention by Scott et al.^35^ showed that practical and theory‐based activities to improve knowledge and skills in interpreting UV and weather forecasts significantly increased the frequency with which participants checked the UVI. In addition, organizations devoted to skin cancer prevention need to take action to reduce the positive image of tanned skin that is still prevalent in the media and in advertising in order to increase the uptake of sun protection measures.^64^ The UVI should play an important role in deciding about suitable sun protection measures, as, contrary to popular belief, the intensity of solar radiation is not highly correlated with the ambient temperature. This means that the UVI can also be very high on clear‐sky days with a moderate temperature during late spring and early summer. Even cloud cover does not necessarily protect against UV radiation. Although clouds can attenuate the radiation through scattering, under certain circumstances the UV radiation on the ground can also be increased by clouds; this effect is known as cloud enhancement.^65^ Of the 14 studies that investigated the active use of the UVI for sun protection, there was one study the results of which were strikingly higher than the proportions reported in the other studies. Busuttil et al.^55^ reported that 78% of the Maltesian population follow the UVI forecast. This study also reported a higher percentage of awareness of the UVI compared to other European studies. However, the reliability of the results of this study remains unclear, as the study results were only published as a poster, which severely limits the information available on the study planning, conduct, and analysis. Consequently, the evaluation of the study results is severely constrained. Overall, the majority of studies in the two outcome subcategories “active use for sun protection” and “actively seeking information about the UVI” had a high ROB (8 out of 14 studies and 6 out of 9 studies, respectively), which makes a bias in the results likely. Only one American and one German study were rated as low ROB, which severely limits the evidence base of high‐quality studies from which reliable conclusions can be drawn about the prevalence of UVI use at the population level in general and any geographical differences in particular. The main reason for a high ROB rating in these studies was a potential selection bias as the study participants were not representative of the target population due to the recruitment methods employed.

As the UVI concept has been devised almost 30 years ago, one would expect that the public awareness and use have increased over time. In the Swiss study by Krebs et al.,^49^ annual surveys of independent study groups were conducted between 2002 and 2008, but the results did not show an increase in awareness over this period. The proportion of people who were aware of the UVI was 37% in 2002 and 38.6% in 2008, with slight fluctuations in between. Nonetheless, the six‐year period analyzed in^49^ is too short to allow general statements about the development of awareness of the UVI over time. Due to the heterogeneity in terms of study region, study methodology, and study population among the studies we identified, it was not feasible to conduct an in‐depth analysis of the temporal development of awareness and use of the UVI. This analysis would require a greater number of studies conducted in the same study regions at different time points.

Our systematic review is not the first to analyze the dissemination, uptake, and use of information about the UVI by the public. In 2012, Italia and Rehfuess^13^ conducted a systematic review trying to answer the question whether the UVI is an effective instrument to promote sun protection. They compiled results from 25 studies and concluded that the UVI “has not been successful at improving sun protection practices and reducing sun exposure among the population at large.” More recently, Heckman et al.^14^ presented the results of a systematic review addressing awareness, understanding, use, and impact of the UVI based on 31 studies published through 2017. Their conclusion was similar to the previous systematic review. In contrast to our systematic review, Heckman et al.^14^ did not critically appraise the methodological quality of the included studies and did not attempt a meta‐analytical summary of the evidence. The results of our systematic review, based on a much larger number of studies than the previous ones (including studies published in the meantime) and performed meticulously according to PRISMA guidance, are consistent with the disappointing findings of the previous systematic reviews and show that the UVI has not yet achieved its intended goals of becoming a global public health tool to promote sun protection.

Raising public awareness of the dangers of UV radiation is a global task. A global project of the WHO called INTERSUN investigates health effects from exposures to UV radiation and recommends health protection measures. The project was introduced in 1995 and encourages countries to take action to reduce health risks from UV radiation and provides guidance to national authorities and other agencies on effective sun awareness programs. Several collaborating centers are involved, including the Federal Office for Radiation Protection (BfS) in Germany, the Australian Radiation Protection and Nuclear Safety Agency in Australia, the Health Security Agency in the United Kingdom, and L'association Securité Solaire in France. Australia has already implemented the recommendations with its SunSmart program even before the start of the INTERSUN project. Other countries have also launched campaigns to promote sun protection. In Germany, for example, the BfS has launched a UV campaign in April 2023 to provide decision‐makers such as local authorities, day‐care centers, schools and sports clubs with information and practical tips on UV radiation and UV protection including use of the UVI.^66^ Nonetheless, the level of knowledge about the UVI among the world's population is still mixed and there is little use of the UVI for personal sun protection. Better integration of the UVI accompanied by sun protection recommendations into weather reports and weather apps may be one way to increase awareness and use.

We have to acknowledge some limitations of this systematic review. Firstly, it is possible that the review may not have included all relevant research on this topic although we have implemented a comprehensive search strategy, which included gray literature sources, in order to minimize the likelihood of overlooking relevant research. Secondly, we had to restrict our meta‐analysis to the outcome “UVI awareness” due to the heterogeneous and occasionally unclear questions posed in the studies that examined the outcome “UVI use.” Furthermore, the meta‐analysis had to be restricted to population‐based studies because of the heterogeneity of the study populations selected in the included studies. A further limitation was the small number of studies with a low ROB, which severely constrained the feasibility of sensitivity analyses. Consequently, it was only possible to conduct the sensitivity analysis for the outcome “general awareness.” Due to the considerable regional influence and the lack of studies conducted at different points in time in a single region, we were not able to address changes in awareness and use of the UVI over time. An implicit limitation of our review arises from the limitation of the individual studies that relied on self‐reports of UVI awareness and UVI use, which were mostly assessed dichotomously (yes/no), corresponding to a rather crude quality of information on these aspects.

## CONCLUSION

Our systematic review revealed a mixed picture of awareness and use of the UVI. While awareness is high in some regions, such as Australia, the use of the UVI is low in all regions studied. To enhance awareness, the UVI must be disseminated with greater efficacy via the media and apps. Nevertheless, the dissemination of a UVI value alone is insufficient; messages tailored to specific exposure situations represent a crucial strategy for rendering the concept more comprehensible and pertinent to people's daily lives. Even almost 30 years after the introduction of the UVI as health promotion instrument, there is a pressing need for increased dissemination of knowledge regarding the risks associated with ultraviolet radiation and the benefits of using the UVI to identify circumstances when sun protection is urgently needed.

## Supporting information


Appendix S1
