# Supplementary material for: The extent of public awareness and use of the Global Solar UV Index as a worldwide health promotion instrument to improve sun protection: A systematic review and meta‐analysis
Source: Photochem Photobiol. 2024 Oct 14;101(3):636–59. doi: 10.1111/php.14028 (PMC12080879; doi:10.1111/php.14028)
Supplement: Supplementary file 1 — Appendix S1 [file PHP-101-636-s001.docx]

# **SUPPORTING INFORMATION**

**The extent of public awareness and use of the Global Solar UV index as a worldwide health promotion instrument to improve sun protection: A systematic review and meta-analysis**

Isabelle Kaiser*^1^, Annette B. Pfahlberg^1^, Maria Lehmann^1^, Esther Buchta^1^, Wolfgang Uter^1^, Olaf Gefeller ^1^

^1^ Institute of Medical Informatics, Biometry, and Epidemiology, Friedrich-Alexander University Erlangen-Nürnberg, Waldstraße 6, 91054 Erlangen, Germany

**Table S1**. Search string for searching the Scopus database

| TITLE-ABS-KEY({UV Index} OR {UVI} OR {Solar Index} OR {Ultraviolet Radiation Index} OR {UVR Index} OR ("UV forecast*") OR ("UV Radiation forecast*") OR {Ultraviolet Index}) |
| --- |
| AND |
| TITLE-ABS-KEY("familiar*" OR "understand*" OR {comprehension} OR {comprehend} OR "know*" OR "aware*" OR {perception} OR {perceive} OR {attitude} OR "behav*" OR {sun tan} OR {suntan} OR {tanning} OR "sunbath*" OR {sunburn} OR "sunscreen*" OR {sunblock} OR {sun protection} OR {midday} OR {noon} OR {dangerous hours} OR {peak hours} OR {sun avoidance} OR {shade} OR "tree*" OR {indoors} OR "cloth*" OR {shirt} OR {sunglasses} OR {shades} OR {hat} OR {sun exposure} OR {time in the sun} OR {sun seeking} OR {use} OR "consider*" OR {Health Belief Model} OR {Protection Motivation Theory} OR {Theory of Reasoned Action} OR {Theory of Planned Behaviour} OR {Theory of Planned Behavior} OR {Transtheoretical Model} OR {Precaution Adoption Process Model} OR {Health Action Process Approach} OR {self-efficacy} OR {belief in efficacy of coping response} OR {perceived behavioural control} OR {perceived behavioral control} OR {plan} OR {planning} OR "intent*" OR {protection motivation} OR {risk perception} OR “perceived threat*” OR {perceived susceptibility} OR {perceived seriousness} OR {appraised severity} OR “fear appeal*”) |
| AND PUBYEAR > 1994 |

**Table S2.** Primary focus of included studies and details on study aims. The studies are sorted in alphabetical order.

| **Author (Year)** | **Primary focus** | **Aim of the study** |
| --- | --- | --- |
| Addas et al. (2021) | Other focus | to analyse UVI data for Saudi Arabia over the period 2004 until 2020 |
| Alberink et al. (2000) | UVI | to investigate Australian adults’ awareness of the UVI forecast in the media, and whether these UV forecasts influence their behavior in the sun |
| Aryal et al. (2018) | Sun protection incl. UVI | to evaluate knowledge and practice regarding UV radiation, photo protection, sunscreens, and sun- related photodermatosis among police officers in Kathmandu |
| Bais et al. (1997) | Other focus | (i) to describe the various steps that have been followed during the last years in Greece for the calculation of the UVI, as well as comparisons of the predicted values with measured data, and (ii) to report prelimnary results about the response of the population on the dissemination of the UVI forecasts |
| Blunden et al. (2004) | UVI | to determine the level of public knowledge relating to the UV index and the relationship between demographic factors, knowledge, and use of the UV index |
| Börner et al. (2010) | UVI | to present representative data on UVI understanding and influence on attitudes towards sun exposure and sun protection behavior in Germany |
| Bränström et al. (2003) | Sun protection incl. UVI | to compare four different UV radiation information packages (including information about the UVI) directed at young adults with respect to their effects on a number of sun-related behaviors |
| Bulliard et al. (2001) | UVI | to assess the public reach, awareness, understanding, and response to the burn time and the UVI in media weather reports in New Zealand |
| Busuttil et al. (2019) | UVI | to assess social trends on the awareness of UVI, sun protection and behavior amongst the Maltese population |
| Capellaro et al. (2015) | Other focus | to evaluate information and early warning systems performed in relation to the degree of penetration, the range and clarity of the offer as well as with regard to the adaptation intentions and the adaptive behavior of the population |
| Carter et al. (2007) | UVI | to evaluate Australians’ understanding of the UVI |
| Diffey et al. (2009) | Sun protection incl. UVI | to gather data about how much time visitors to the SunSmart website spend in the sun, their preferred forms of sun protection and their use of tools such as sun-reactive skin type and UVI |
| Gao et al. (2014) | Sun protection incl. UVI | to assess the knowledge, attitudes, and practices regarding the health effects of UV radiation and sun exposure among medical university students in Northeast China |
| Gefeller et al. (2022) | UVI | to assess awareness of, and knowledge about, the UVI as well as the consequences of UVI information for daily sun protection |
| Geller et al. (1997) | UVI | to evaluate the extent to which television stations and newspapers reported the UVI and assess the public's response to it |
| Government Statistical Service (2000) | UVI | to report results about people’s knowledge and awareness of the UVI in Great Britain |
| Harrison et al. (2007) | Sun protection incl. UVI | to provide data about the sun-protection knowledge of early childhood directors/senior staff, the adequacy of sun-protective behaviors practiced by children and staff members at each service and the proportion of Queensland-based services with sun-protection policies |
| Hault et al. (2016) | Sun protection incl. UVI | to assess the knowledge of workers in outdoor professions on the effects of natural UV radiation and methods of protection against exposure |
| Klostermann et al. (2014) | Sun protection incl. UVI | to identify determinants of parental sun protection behavior |
| Krebs et al. (2008) | UVI | to investigate awareness and use of the UV Index among the population of German- and French-speaking Switzerland. |
| Kricker et al. (1997) | UVI | to answer the following questions:  • How regularly, widely, and in what form are the retrospective and prospective indexes being disseminated by the news media? • What do members of the general public know about the UV indexes, how frequently do they observe them, what use do they make of them in determining their outdoor exposure and protection, and how could they be made more useful to them? • What do health professionals substantially involved in communications with the public about sun protection know about the UV indexes, what use do they make of them, their communication with the public, how useful do they think they are to the public and how could they be made more useful? |
| Mair et al. (2012) | Sun protection incl. UVI | to assess the willingness of young adults to receive electronic messages to improve their sun protection behaviors, and to identify factors associated with their interest in receiving such messages |
| Makin et al. (2007) | UVI | to determine levels of awareness, understanding, and use of the SunSmart UV Alert among the general public in Victoria |
| McCarthy et al. (1999) | Sun protection incl. UVI | to estimate the frequency of sunburn among the beach-going population at a popular public beach on Galveston Island during a national summer holiday weekend and to address the level of knowledge, use of topical sunscreen and other sun-protective measures in relation to ultraviolet light exposure, sunburn, and skin cancer among beach-goers. |
| McGee et al. (2002) | Sun protection incl. UVI | to examine patterns and correlates of sun protection among young children at beaches and playgrounds in Dunedin (New Zealand) and beaches in Hawkes Bay, using a combination of direct observation and parent/carer interview |
| Morales-Sánchez et al. (2021) | Other focus | to determine the prevalence of sun exposure and sun protection behaviors in a group of adolescents and compare them with a group of adults living in Mexico City |
| Morris et al. (2011) | UVI | to explore awareness and understanding of the UVI information presented in weather forecasts; and to determine whether individuals change their sun exposure/protection behavior as a result of receiving such information |
| Patlola et al. (2023) | Other focus | to assesses the general knowledge of US citizens on sunscreen use, frequency of usage, common behaviors of sunscreen usage, and use of sunscreen based on knowledge of sun protection |
| Purdue et al. (2001) | Other focus | to provide information about sun exposure and protective behaviors in the Canadian population |
| Reeder et al. (2001) | Sun protection incl. UVI | to examine the public reach, awareness, understanding and response to sun protection messages in media weather reports in New Zealand (Maori participants) |
| Scott et al. (2021)^†^ | Sun protection incl. UVI | to investigate primary preservice teachers current tanning and sun protection behaviors, awareness of the dangers related to UV exposure and perceived knowledge and skill to be able to teach sun safety |
| Scott et al. (2021)^‡^ | Sun protection incl. UVI | to investigate the effect of a short intervention on preservice teachers sun protective behaviors, attitudes, perceived knowledge, and understanding of UV and also their confidence and skills to be able to teach sun safety in primary school settings |
| Sécurité Solaire (2000) | UVI | to investigate awareness and understanding of the UV Index disseminated by Sécurité Solaire, as well as changes in the public's level of information about the health risks associated with exposure to the sun, and the impact of the UV Index. |
| Sin et al. (2013) | UVI | to assess dermatologists' knowledge of UVI in mainland France, and their personal and professional use of this tool. |
| Sultana et al. (2020) | Sun protection incl. UVI | to estimate the level of sun protection practices and determine the factors that have a significant impact on the level of adoption of sun protection practices by residents in the Arabian Peninsula |
| Thomas et al. (2017) | Sun protection incl. UVI | to assess skin cancer and sun safety knowledge and behaviors in adults seeking a skin check |
| Unverricht et al. (2007) | Sun protection incl. UVI | to investigate individual behavior and the state of basic knowledge regarding the handling of solar UV radiation |
| Wester et al. (2000) | UVI | to evaluate the influence of the UV index and the UV forecasts on the sun exposure habits of people in Sweden |
| White et al. (1997) | UVI | to examine the awareness of UV forecasts amongst the general public, action taken in response to UV information, and understanding of UV forecasts |
| Wright et al. (2014) | Sun protection incl. UVI | to describe the sun-related knowledge, attitudes and behaviors as self-reported by South African primary schoolchildren |

^†^ Scott, J. J., R. S. Johnston, N. Bear, S. Gregory, S. Blane and M. Strickland (2021) Targeted Teacher Education to Improve Primary Preservice Teachers’ Knowledge and Understanding of UV and Effective Sun Protection Measures for Children. Australian Journal of Teacher Education 46, 51-73.
^‡^ Scott, J. J., R. Johnston, S. Blane, M. Strickland, J. Darby and E. Gray (2021) Investigating primary preservice teachers' ultraviolet radiation awareness and perceived ability to teach sun safety. Health Promot J Austr 32 Suppl 2, 178-184.


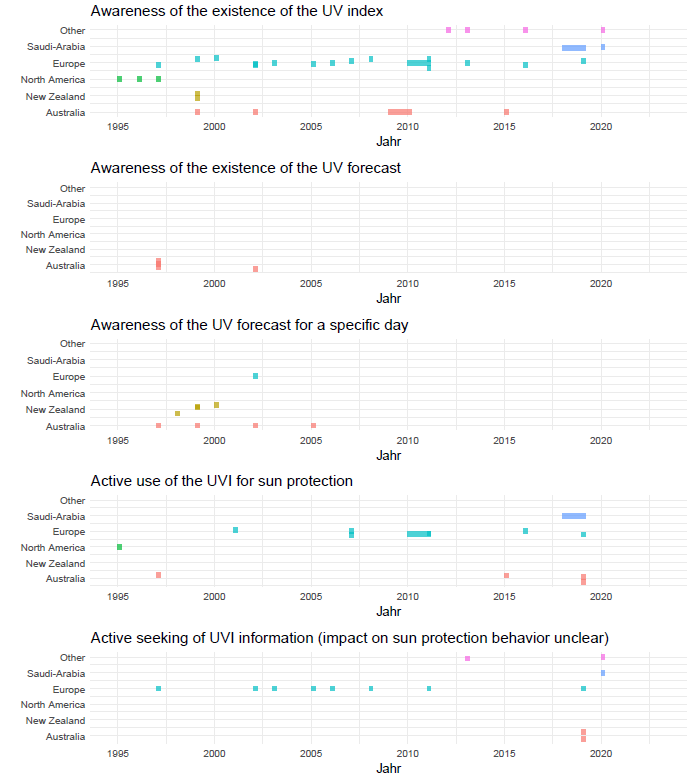


**Figure S1.** Temporal distribution of data collection periods of included studies, stratified by outcome and region. The different colors represent the different study regions (Australia: red; New Zealand: yellow; North America: green; Europe: turquoise, Saudi-Arabia: blue; Other: purple).


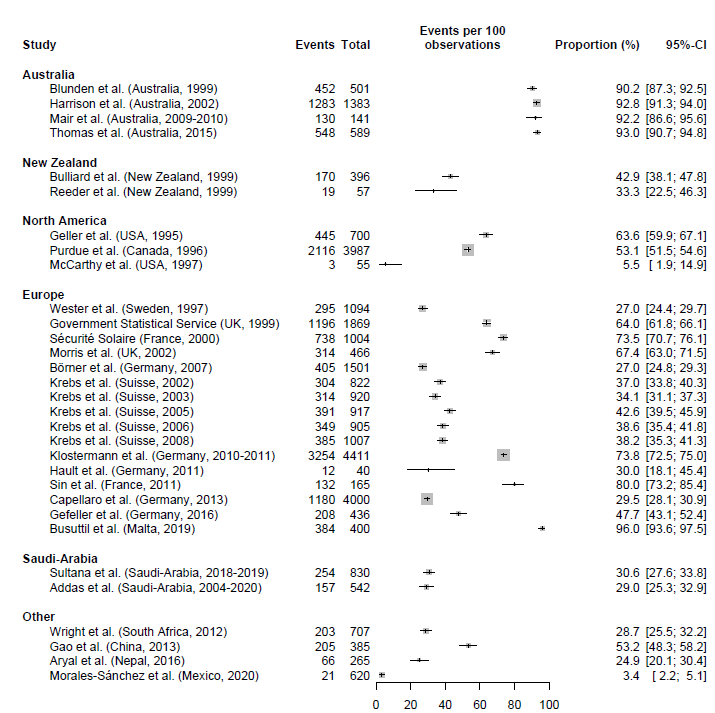


**Figure S2.** Forest plot showing the proportion of general awareness of the UVI for all 27 studies that examined the outcome. Studies are grouped by study region and within each region sorted according to the period of data collection. The size of the box is proportional to the sample size.


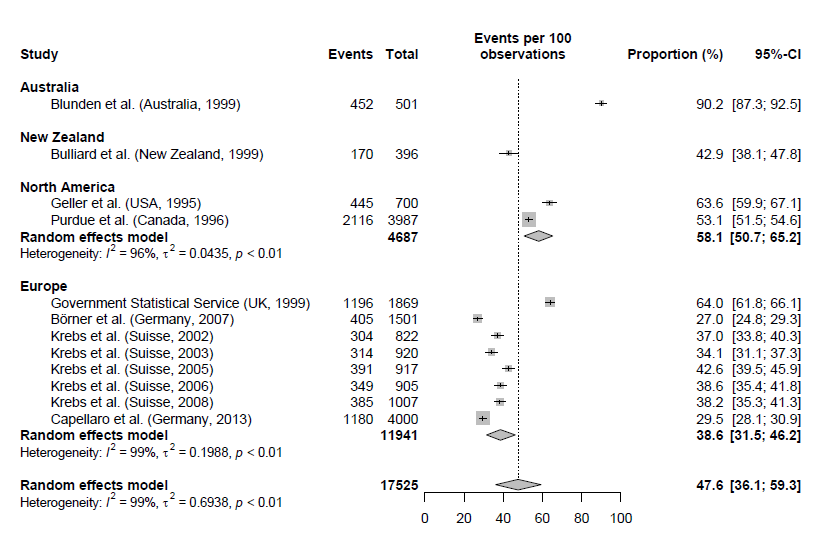


**Figure S3.** Results of sensitivity analysis including only population-based studies rated as having a low ROB. Studies are grouped by study region and within each region sorted according to the period of data collection. The size of the box is proportional to the sample size.
